# Fifth Commencement of the Chattanooga Medical College

**Published:** 1894-04

**Authors:** 


### FIFTH COMMENCEMENT OF THE CHATTANOOGA MEDICAL COLLEGE.

The commencement exercises of this college were held in the opera house, in that city, March 27th. The class of graduates, which were forty-nine in number, was the largest in the history of the college.

Dr. J. R. Rathmell, the orator of the evening, delivered a fine address, in which he took occasion to say many kind words of encouragement to the young physicians.

Dr. J. R. Peace, the valedictorian, made a fine address and was highly complimented on his effort.

Following the announcement of the names of the class, Dr. Rathmell announced that the honors had been won by the following gentlemen: first honor, Dr. J. A. McCollum; second, Dr. J. M. Clark; third, Dr. A. B. Gillihand. The following gentlemen received honorable mention: Dr. M. P. Bates, Dr. D. S. Middleton, Dr. W. K. Burnett and Dr. H. Chisholm.

---

